# Post-marketing safety surveillance for both CRM197 and TT carrier proteins PCV13 in Jiangsu, China

**DOI:** 10.3389/fpubh.2023.1272562

**Published:** 2023-10-16

**Authors:** Ran Hu, Yuanbao Liu, Lei Zhang, Guodong Kang, Borong Xu, Mingma Li, Jing Yu, Yuanyuan Zhu, Hongxiong Guo, Zhiguo Wang

**Affiliations:** ^1^Jiangsu Provincial Center for Disease Control and Prevention, Nanjing, China; ^2^Department of Epidemiology and Health Statistics, Southeast University School of Public Health, Nanjing, China

**Keywords:** vaccine, pneumococcal polysaccharide conjugate vaccine, adverse reaction, carrier protein, safety, post-marketing surveillance

## Abstract

**Background:**

This study is to evaluate the safety of two kinds of PCV13 carriers by monitoring the occurrence of adverse event following immunization (AEFI) after the launch of two kinds of PCV13 carriers in Jiangsu Province, China.

**Methods:**

The AEFI Information System (CNAEFIS) of mainland China was used to monitor the incidence and classification of adverse reactions of the CRM197-carrier protein PCV13 and TT-carrier protein PCV13 vaccines.

**Results:**

There was no statistical difference between the cumulative reported incidence of AEFI between the two vaccines from 2020 to 2022 (*χ*^2^ = 1.991, *p* < 0.158). 96.62% of the AEFIs were classified as common reactions; rare reactions and coincidental events only accounted for 2.99 and 0.39% of all the AEFI cases, respectively. Redness (2.6 cm–5 cm) is the commonest symptom at the injection site for both vaccines. More than 97% of AEFIs occurred between 30 min and 3 days after administration for both types of PCV13.

**Conclusion:**

Both vaccines perform well in terms of safety. We did not identify any new/unexpected safety concern from the NAEFISS during a 4 years timespan.

## 1. Introduction

*Streptococcus pneumoniae* is the most common cause of community-acquired pneumonia, bacterial meningitis, bacteremia, and otitis media. As normal flora of human upper respiratory tract of human, it is often found in the nasopharynx of healthy people, and about 20 to 50% of people carry them (1, 2). When a human body lies under immunocompromising conditions, colonized pneumococcal is likely to develop into invasive disease (2). In recent years, pneumococcus has developed varying degrees of resistance to antibiotics, making the treatment of pneumonia more difficultly (3). In 2019, there were an estimated 13.7 million infection-related deaths globally of which more than 500,000 deaths were associated with pneumococcal infections (4, 5). Among the countries with the highest burden of pneumococcal diseases in children under 5 years of age, China ranks 4th,which accounts for about 3% of the total number of cases in the world (4, 6). Considering the infection rate, morbidity, mortality rate, the resistance of Streptococcus pneumonia, and cost-effectiveness of prevention and treatment of the disease, there is an urgent need to control pneumococcal disease using vaccines (3, 6–8).

The World Health Organization (WHO) recommends that all countries, especially those with a mortality rate of >50‰ for children under 5 years old, include the pneumococcal conjugate vaccine (PCV) in their National Immunization Programs (NIP) (9). As of 2023, China has not integrated PCVs into her NIP (10). At present, there are two kinds of products of the 13-valent pneumonia conjugate vaccine (PCV13) worldwide. One is PCV13 conjugated with cross-reactive material 197 (CRM197), and the other is PCV13 conjugated with tetanus toxoid (TT). The latter is China’s first PCV-13, which has been licensed by China National Medical Products Administration (NMPA) in December 2019 for all children aged 2 months to 5 years (4).

In view of the high number of serotypes and complex mechanism of PCV vaccines, manufacturers have been looking for suitable carrier proteins and combinations. Before the launch of TT-carrier protein PCV13, there were some doubts about the safety of the vaccine with TT as the carrier protein (11). According to the previous researches on TT, this carrier protein is the inactivated tetanus toxin manipulated with formaldehyde, which may suffers from formaldehyde residuals and the incomplete detoxification of tetanus toxin in vaccine products (11, 12). In other words, TT carrier protein is an inactivated tetanus toxin, whose safety depends on whether it is inactivated completely during production process. TT-based conjugate vaccine may show high rates of irritability, crying, and fever (11). Therefore, whether the post TT-carrier protein PCV13 is really affected by its carrier protein is still unclear. The study aimed to assess the safety of PCV13. Specifically, a comparison was conducted between the CRM197-carrier protein PCV13 and the TT-carrier protein PCV13. This study might help policy-makers in their decisions to continue adjusting its vaccination schedule or integrating it into NIP.

## 2. Methods

### 2.1. Vaccination schedule for PCV13

The CRM197-carrier protein PCV13 was administered to children aged from 6 weeks to 15 months old, whereas the TT-carrier protein PCV13 was administered to children aged from 6 weeks to 5 years old. Both kinds of PCV13 vaccines were administered via the intramuscular route using a four-dose schedule. But TT-carrier protein PCV13 immunization schedule is more flexible. Children aged 7–11 months can be vaccinated with 2 doses and reinforced immunization with 1 dose after 12 months. Children aged 12–23 months can be vaccinated with 2 doses, with an interval of at least 2 months. Children aged 2–5 years can be vaccinated with 1 dose.

### 2.2. Surveillance of AEFI

In mainland China, a passive adverse event following immunization(AEFI) Information System (CNAEFIS) to be used for AEFI monitoring was established in 2008, and updated twice in 2015 and 2018, respectively. Through this system, doctors of VCs (vaccination clinics) with authority report and preliminary classify AEFI. This system has been described in our previous research (13). The definition of adverse event following immunization in Mainland China is similar to the requirements of WHO National Regulatory Authority (NRA) assessment. AEFI refers to the reaction that may cause the damage of organs and functions of the person affected in the course of preventive inoculation or after inoculation, and suspected to be related to preventive inoculation.

### 2.3. Information on AEFIs of PCV13

AEFIs occurring after the administration of the PCV13 in Jiangsu Province from January 1st 2019 to December 31st 2022 were reviewed. AEFI data that were extracted from the CNAEFIS during the above period were included in this study for cases in which any type or dose of PCV13 was administered. We obtained the unique identification code, gender, age, inoculation time, reaction time, preliminary AEFI classification, final clinical diagnosis, final AEFI classification and other information of the recipients with AEFI after PCV13 inoculation in the selected time period from CNAEFIS.

### 2.4. Number of PCV13 doses

The Chinese individual-level Electronic Immunization Registries System (EIRS) has already been introduced in our team’s previous research. Jiangsu has developed a new system (Jiangsu Province Vaccination Integrated Service Management Information System, SMIS) on the basis of that system. The name, age, sex, inoculation record, place of residence and other information of the recipient can be queried in detail and summarized statistically in SMIS. With this new system, the number of PCV13 doses was obtained and selected as a denominator to calculate the IR of the AEFI.

### 2.5. Statistical analysis

The differences of AEFI occurrence between two kinds of vaccines on gender, doses, year of AEFI reporting were analyzed by chi-square test with R package. Poisson test was performed on the distribution of various AEFI proportion of both PCV13 vaccines. In this study, the Northern Jiangsu includes Xuzhou, Huai’an, Lianyungang, Suqian, Yancheng; the Central Jiangsu includes Nantong, Yangzhou, Taizhou; the Southern Jiangsu includes Nanjing, Zhenjiang, Changzhou, Wuxi, Suzhou. The incidence rate of an AEFI (per 100,000 doses) is the reported number of AEFI per 100,000 doses administrated.

## 3. Results

### 3.1. Baseline data

During the period from January 1, 2019 to December 31, 2022, a total of 1,915,958 doses of PCV13 were registered in Jiangsu Province, China, of which 1,439,808 doses of CRM197-carrier protein vaccine were inoculated, accounting for 75.1%; 476,150 doses of TT-carrier protein vaccine were administered, accounting for 24.9%. 2,726 AEFI cases linked to CRM197-carrier protein PCV13 and 853 linked to TT- carrier protein PCV13 were reported in CNAEFIS, respectively.

The AEFI IR sex ratio of both PCV13 were 1:0.92, while the difference was not statistically significant (*χ*^2^ = 2.35, *p* = 0.125). There was no difference in the incidence of AEFI among different vaccines after gender stratification was taken into account (CMH*χ*^2^ = 1.938, *p* = 0.164). The IR of AEFIs in 2020 was higher than that in other years (*χ*^2^ = 31.282, *p* < 0.001). There was no significant difference in the incidence of AEFI among different carrier protein vaccines after considering the influence of year stratification factors (CMH*χ*^2^ = 0.028, *p* = 0.868). There were significant differences in the incidence of AEFI among various regions in Jiangsu Province. The incidence of AEFI in northern Jiangsu Province was significantly higher than that in central and southern areas (*χ*^2^ = 4780.58, *p* < 0.001). Among children received the fourth dose of vaccine, the incidence of AEFIs is higher than in those only received the first three doses (*χ*^2^ = 120.81, *p* < 0.001). We compared the occurrence of AEFI among different recipient groups, and found that there was significant difference between the first dose and the third dose (*χ*^2^ = 5.50, *p* = 0.019) while no significant difference between the second dose and the third dose (*χ*^2^ = 0.20, *p* = 0.653). There was no statistical difference between the cumulative reported incidence of AEFI between the two vaccines from 2020 to 2022 (*χ*^2^ = 1.991, *p* < 0.158) (shown in Table 1).

**Table 1:** Basic distribution characteristics of PCV13 AEFI in Jiangsu Province from 2020 to 2022.

Variable		CRM197-Carrier protein			TT-Carrier protein			Total			*χ* ^2^	*p*	CMH*χ*^2^	CMH *p*
		Vaccine doses	AEFI cases	IR	Vaccine doses	AEFI cases	IR	Vaccine doses	AEFI cases	IR				
Sex	Male	746,339	1,486	199.11	244,138	482	197.43	990,477	1968	198.69	0.026	0.872	1.938	0.164
	Female	693,469	1,240	178.81	232,012	371	159.91	925,481	1,611	174.07	3.576	0.059		
Year	2019	312,260	581	186.06	–	–	–	312,260	581	186.06	–	–	0.028	0.868
	2020	408,537	897	219.56	13,421	21	156.47	421,958	918	217.56	2.383	0.123		
	2021	366,058	644	175.93	162,776	250	153.59	528,834	894	169.05	3.333	0.068		
	2022	352,953	604	171.13	299,953	582	194.03	652,906	1,186	181.65	4.691	0.030		
Dose	First Dose	395,509	744	188.11	191,340	345	180.31	586,849	1,089	185.57	0.424	0.515	0.535	0.464
	Second Dose	380,411	583	153.26	137,359	278	202.39	517,770	861	166.29	14.676	<0.001		
	Third Dose	364,073	581	159.58	90,941	139	152.85	455,014	720	158.24	0.209	0.648		
	Fourth Dose	299,815	818	272.83	56,510	91	161.03	356,325	909	255.1	23.357	<0.001		
Place of residence	Northern Jiangsu	264,195	2005	758.91	161,153	467	289.79	425,348	2,472	581.17	381.257	<0.001	159.283	<0.001
	Central Jiangsu	191,659	346	180.53	83,706	162	193.53	275,365	508	184.48	0.535	0.464		
	Southern Jiangsu	983,954	375	38.11	231,291	224	96.85	1,215,245	599	49.29	131.139	<0.001		
Total		1,439,808	2,726	189.33	476,150	853	179.15	1,915,958	3,579	186.80	1.991	0.158		

IR, incidence rate per 100,000 vaccine doses.

### 3.2. AEFIs symptoms

96.62% of the AEFIs belongs to common reactions; rare reactions and coincidental events only accounted for 2.99 and 0.39% of all the AEFI cases, respectively (Table 2). No vaccine quality events, program errors, psychogenic reactions, or deaths were reported. Among the individuals with AEFI in all years, there was a statistical difference in the proportion of various types of AEFIs caused by these two vaccines (*χ*^2^ = 17.32, *p* = 0.0002).

**Table 2:** CRM197-Carrier protein and TT-Carrier protein classification of AEFIs.

The classification of AEFIs	CRM197-Carrier protein		TT-Carrier protein		*χ* ^2^	*p* value
	AEFI cases	IR (per 100,000 doses)	AEFI cases	IR (per 100,000 doses)		
Common reaction	2,615	181.62	843	177.05	17.32	0.0002
Rare reaction	97	6.74	10	2.1		
Coincidental event	14	0.97	0	0		
Total	2,726	189.33	853	179.15		

A total of 2,726 AEFIs linked to the CRM197-carrier protein PCV13 were reported, in which 1,390 were injection site reactions. The IRs of different symptoms ranged from 0.07 to 70.15 (per 100,000 doses). The top three systemic symptoms were fever, irritability and loss of appetite (Table 3).

**Table 3:** CRM197-Carrier protein and TT-Carrier protein systemic reaction and injection site reactions.

AEFIs symptoms		CRM197-Carrier protein		TT-Carrier protein	
		Number (%)	IR (per 100,000 doses)	Number (%)	IR (per 100,000 doses)
Systemic reaction	Fever (>38.6°C)	755 (27.70)	52.44	169 (19.81)	35.49
	Fever (37.6°C–38.5°C)	1,010 (37.05)	70.15	187 (21.92)	39.27
	Fever (37.1°C–37.6°C)	147 (5.39)	10.21	36 (4.22)	7.56
	Irritability	529 (19.41)	36.74	225 (26.38)	47.25
	Allergic eruption	84 (3.08)	5.83	11 (1.29)	2.31
	Fatigue	99 (3.63)	6.88	35 (4.10)	7.35
	Loss of appetite	118 (4.33)	8.2	37 (4.34)	7.77
	Drowsiness	76 (2.79)	5.28	12 (1.41)	2.52
	Vomiting	28 (1.03)	1.94	12 (1.41)	2.52
	Diarrhea	27 (0.99)	1.88	7 (0.82)	1.47
	Pruritus	33 (1.21)	2.29	29 (3.40)	6.09
	Cough	10 (0.37)	0.69	0 (0.00)	0
	Sweat	8 (0.29)	0.56	6 (0.70)	1.26
	Rhinorrhea	0 (0.00)	0	0 (0.00)	0
	Skin pale	1 (0.04)	0.07	0 (0.00)	0
	Abdominal pain	0 (0.00)	0	0 (0.00)	0
Injection site reactions	Redness(>5 cm)	252 (9.24)	17.5	172 (20.16)	36.12
	Redness(2.6 cm–5 cm)	504 (18.49)	35	281 (32.94)	59.02
	Redness(≤2.5 cm)	197 (7.23)	13.68	76 (8.91)	15.96
	Induration(>5 cm)	59 (2.16)	4.1	62 (7.27)	13.02
	Induration(2.6 cm–5 cm)	215 (7.89)	14.93	128 (15.01)	26.88
	Induration(≤2.5 cm)	163 (5.98)	11.32	82 (9.61)	17.22
Total		2,726	189.33	853	179.15

A total of 853 AEFIs linked to the TT-carrier protein PCV13 were reported, in which 801 were injection site reactions. The IRs of different symptoms ranged from 0 to 59.05 (per 100,000 doses) for the TT-carrier protein PCV13. The top three systemic symptoms were fever, irritability and loss of appetite. Redness (2.6 cm–5 cm) is the commonest symptom at the injection site for both vaccines (Table 3).

### 3.3. AEFIs time intervals

More than 97% of AEFIs occurred between 30 min and 3 days after administration for both kinds of PCV13 (Table 4). The main symptoms with a time interval of less than 30 min were allergy related symptoms, mainly allergic rash. The longest interval was 17 days. The clinical symptom of this case was thrombocytopenic purpura after vaccination. The recipient’s guardian believed that it was caused by vaccine. However, the AEFI was classified as a coincidental event after the investigation of the AEFI expert panel.

**Table 4:** Compare of CRM197-Carrier protein and TT-Carrier protein AEFIs time intervals.

AEFIs time intervals	CRM197-Carrier protein		TT-Carrier protein		Total		*χ* ^2^	*p* value
	AEFI cases (%)	IR (per 100,000 doses)	AEFI cases (%)	IR (per 100,000 doses)	AEFI cases	IR (per 100,000 doses)		
≤30 min	32 (1.17)	2.22	9 (1.06)	1.89	41 (1.15)	2.14	5.20	0.0743
30 min–3 days	2,662 (97.65)	184.89	825 (96.72)	173.26	3,487 (97.43)	182		
>3 days	32 (1.17)	2.22	19 (2.23)	3.99	51 (1.42)	2.66		
Total	2,726	189.33	853	179.15	3,579	186.80		

## 4. Discussion

Widespread use of 23-valent pneumococcal polysaccharide vaccine (PPV23) and PCV13 has significantly decreased invasive pneumococcal disease (IPD) in children (14, 15). The significant reduction in the burden of pneumococcal disease in children worldwide over the last decade underscores the public health value of infant immunization with pneumonia vaccine (16–18). Although the 13-valent pneumonia vaccine is not included in China’s National Expanded Programme on Immunization, Chinese parents are highly motivated to vaccinate their children against pneumonia.

Previous study shows that no significant differences on AEFIs were observed between PHiD-CV + MenC-TT and PHiD-CV + MenC-CRM, which is accordance with our results on PCV13-CRM197 and PCV13-TT (19). In addition, our results showed that the reported incidence of AEFI associated with these two kinds of vaccines was higher than that of many other vaccines reported in the national AEFI monitoring system (20). As one of the most expensive category II vaccines for children to be vaccinated at present, which is mainly administrated among children whose parents with good economic status and educational level (4, 21, 22). These parents often pay more attention to the situation of their children after vaccination than others, accompanying with the increasing active reporting rate and sensitivity of AEFI.

We observed no significant difference in reported AEFI incidence between boys and girls, which is generally consistent with previous reports (23, 24). We found that the reported incidence of AEFI in 2020 was highest from 2019 to 2022. Since February 2021, population-wide immunization of COVID-19 vaccine programme was carried out, more efforts and manpower were put in to strengthen AEFI surveillance linked to COVID-19 vaccination in many provinces. It could cause indirectly that the reported incidence of AEFIs except COVID-19 vaccine declined, and this phenomenon lasted from 2021 to 2022. Nonetheless, the reduction in reported AEFIs was mainly focused on general response induced by vaccine. Our results indicated that the reported incidence of AEFI after inoculating the fourth dose of PCV13 is highest whatever for CRM197 or TT conjugated vaccine. Which is distinct from some previous studies which showed that the reported incidence of AEFI after inoculating the first dose is highest (25, 26). Their argument is the recipients and their guardians pay more attention to the reaction after the first dose of vaccine (25, 26). However, there are some studies that support our conclusions (24, 27). The view they take is the subsequent dose was more likely to cause allergic reactions due to the body sensitization by the early dose. No significant difference on the reported incidence of AEFIs was observed among three geographical regions in Jiangsu Province, which was in accordance with our previous studies (13, 25).

AEFI in PCV13 occurs mainly within 2 days after vaccination and generally lasts about 72 h in previous studies (23–25). This is basically consistent with the conclusion of our results. The main clinical symptoms of AEFI caused by PCV13 are general reactions such as fever, redness or scleroma, which usually occur in 2–5 days after vaccination. Guardians’ awareness of reporting AEFI often wanes over time. Even though some minor adverse reactions occur after 5 days, they are often neglected by guardians because they think it is only mild presentation. Similar phenomena have been observed in post-marketing studies of other vaccines. This is actually one of the drawbacks of passive monitoring (21).

There are three limitations to this study. (1) Recipients have different tolerance for adverse events from domestic vaccine (developed and produced in China) and imported one (developed and produced outside China). Some recipients may subconsciously believe that imported vaccines have fewer adverse effects. Even if there is an adverse reaction, they get used to attribute it to personal physique. However, we do not think it will have a significant impact on the conclusions given the nearly 2 million doses. (2) Reporting bias and information bias cannot be well controlled, for the passive monitoring system. This may affect the accuracy of the results to some extent. Active surveillance study in the future may be a way to solve this problem. (3) The AEFI data associated with TT-carrier protein PCV13 in 2019 was unavailable. So we compare reported AEFI incidence between various years, only the data collected during 2020–2022 were used to conduct statistical analysis.

In conclusion, our findings demonstrated that most AEFIs caused by both PCV13 vaccines were mild and belonged common reactions. No significant differences in rare reactions were observed between TT-carrier protein PCV13 vaccine and CRM197-carrier PCV13. Which was broadly consistent with the safety data from the previous pre- or post-licensure trials. Our results is helpful to reduce the concern from parents for the safety of TT-carrier protein PCV13 vaccine and increase the coverage of PCV13 vaccine in Chinese children.

## Data availability statement

The raw data supporting the conclusions of this article will be made available by the authors, without undue reservation.

## Author contributions

RH: Conceptualization, Data curation, Formal analysis, Writing – original draft, Investigation, Supervision. YL: Investigation, Methodology, Data curation, Software, Visualization, Validation, Writing – review & editing. LZ: Data curation, Methodology, Resources, Writing – review & editing. GK: Investigation, Project administration, Validation, Data curation, Formal analysis, Writing – review & editing. BX: Investigation, Methodology, Validation, Visualization, Writing – review & editing. ML: Data curation, Methodology, Formal analysis, Investigation, Writing – review & editing. JY: Data curation, Methodology, Investigation, Visualization, Writing – review & editing. YZ: Investigation, Data curation, Validation, Visualization, Writing – review & editing. HG: Data curation, Conceptualization, Formal analysis, Writing – original draft, Writing – review & editing. ZW: Data curation, Conceptualization, Methodology, Writing – original draft.
